# Guidelines to Evaluate Human Observational Studies for Quantitative Risk Assessment

**DOI:** 10.1289/ehp.11530

**Published:** 2008-08-12

**Authors:** Jelle Vlaanderen, Roel Vermeulen, Dick Heederik, Hans Kromhout, European Union Network of Excellence ECNIS Integrated Risk Assessment Group

**Affiliations:** Utrecht University, Institute for Risk Assessment Sciences, Division Environmental Epidemiology, Utrecht, the Netherlands

**Keywords:** benzene, epidemiology, evidence-based medicine, human observational studies, quantitative risk assessment

## Abstract

**Background:**

Careful evaluation of the quality of human observational studies (HOS) is required to assess the suitability of HOS for quantitative risk assessment (QRA). In particular, the quality of quantitative exposure assessment is a crucial aspect of HOS to be considered for QRA.

**Objective:**

We aimed to develop guidelines for the evaluation of HOS for QRA and to apply these guidelines to case–control and cohort studies on the relation between exposure to benzene and acute myeloid leukemia (AML).

**Methods:**

We developed a three-tiered framework specific for the evaluation of HOS for QRA and used it to evaluate HOS on the relation between exposure to benzene and AML.

**Results:**

The developed framework consists of 20 evaluation criteria. A specific focus of the framework was on the quality of exposure assessment applied in HOS. Seven HOS on the relation of benzene and AML were eligible for evaluation. Of these studies, five were suitable for QRA and were ranked based on the quality of the study design, conduct, and reporting on the study.

**Conclusion:**

The developed guidelines facilitate a structured evaluation that is transparent in its application and harmonizes the evaluation of HOS for QRA. With the application of the guidelines, it was possible to identify studies suitable for QRA of benzene and AML and rank these studies based on their quality. Application of the guidelines in QRA will be a valuable addition to the assessment of the weight of evidence of HOS for QRA.

Epidemiologic evidence is the most relevant type of evidence for risk assessment, because limited extrapolation is needed to apply study results to a real-life situation. However, because of ethical considerations epidemiologic assessment of risk of potential hazardous exposures is most often limited to observational studies. This deviation from experimental study conditions (e.g., randomized clinical trials) requires careful evaluation of the quality of the observational evidence. A major issue in human observational studies (HOS) is the more limited control of circumstances under which studies are performed leading to a potential bias in the estimated association between exposure and health outcome. The quality of the design and conduct of a study affects the potential for bias in the study results and thus the value for risk assessment. In quantitative risk assessment (QRA), exposure–response relations are defined in quantitative terms (i.e., risk per unit of exposure). HOS that conducted quantitative exposure–response analysis (i.e., a quantitative description of the relation between exposure to a hazardous agent and a specific health effect) can contribute directly to QRA. Therefore, the quality of quantitative exposure assessment is crucial to HOS used in QRA. In recent years, several frameworks have been developed to assess the quality of HOS for risk assessment [Goldbohm et al. 2006; Hertz-Picciotto 1995; Money and Margary 2002; Shore et al. 1992; Swaen 2006; van den Brandt et al. 2002; World Health Organization (WHO) Working Group 2000]. These frameworks have provided broad overviews of different aspects that contribute to HOS quality. However, the existing frameworks lack a specific focus on the evaluation of exposure assessment in HOS for QRA. We developed a structured framework with guidelines for the evaluation of HOS in QRA that have a specific focus on the evaluation of the exposure assessment component of HOS. The approach incorporates exclusion of HOS that do not meet the minimal quality required for QRA and ranking based on the quality of the design, conduct, and reporting of the HOS that do meet the minimal quality required for QRA. Subsequently, to demonstrate its usefulness, we applied the framework to all case–control and cohort studies on the relation between exposure to benzene and acute myeloid leukemia (AML).

## Definition of Terms Related to Quantification of Exposure in QRA

The exposure evaluation guidelines are related largely to the assessment and assignment of exposure. Exposure assessment is defined as estimation of the concentration of an agent in a specific medium (e.g., air or soil) during a specific time period (e.g., a working day) and under specific conditions (e.g., type of weather) (Zartarian et al. 1997). Examples are the concentration of respirable crystalline silica to which a worker was exposed in his breathing zone on a specific day performing a specific task, or the level of caffeine in a single cup of coffee. The most direct strategy for exposure assessment is to perform quantitative measurements. However, in many HOS, exposure measurements are scant, and other sources of information (e.g., expert judgment, questionnaire data, or predictive models) are used to assess exposure (Ott 2005). Exposure assignment is defined as the step where exposure estimates are assigned to the individuals in the study population based on information on, for instance, jobs held or food frequency questionnaires (Loomis and Kromhout 2004).

## Description of the Framework and Evaluation Guidelines

**Figure 1:**
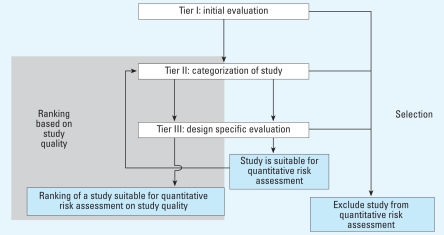
Decision pathway of the framework for evaluation of HOS for QRA. Outcomes of the pathway: exclude study from QRA; study is suitable for QRA; and ranking of a study suitable for QRA based on study quality.

The criteria that together form the guidelines for evaluation of HOS for QRA are described in detail in the Supplemental Material [see Supplemental Material I, Evaluation Guidelines (http://www.ehponline.org/members/2008/11530/suppl.pdf) for details]. Here we provide an overview of the structure of the framework and discuss the evaluation criteria that are crucial for the quality of the assessment and assignment of exposure. The framework is based on three tiers (Figure 1). The criteria in the first tier are used to exclude studies not suitable for QRA and should be applied to all HOS considered for QRA (Table 1). The questions in tier I are all related to crucial aspects of the quality of the design, the quality of conduct, and the quality of the reporting of HOS. Therefore, HOS are suitable for QRA only if all questions are answered affirmatively. A negative answer to one of the questions should result in exclusion of the HOS for QRA.

In the second tier, the HOS are categorized based on the type of study design (Table 1). The reason for categorization in tier II is 2-fold: exclusion of HOS that have an inappropriate study design for QRA, and selection of appropriate criteria for further evaluation in tier III. In the third tier, a decision is made whether to include HOS in QRA based on a set of design specific criteria. A distinction is made between the criteria intended to assess whether HOS are suitable for QRA and the criteria intended to be used in ranking of the HOS suitable for QRA based on the quality aspects of these HOS. Some criteria in Table 1 are used in both the selection and ranking of HOS. Although this framework has been developed primarily to facilitate objective evaluation of HOS for QRA, the criteria in the framework can also be used as guidelines for the conduct of high-quality HOS suitable for future QRA. To facilitate transparent and objective evaluation of evidence from HOS, risk assessors should *a priori* define minimum requirements for including a study in QRA, such as *a priori* definitions of acceptable levels of the response rate and loss to follow-up. In addition, the minimal follow-up time required to detect the health effect of interest should be defined. Finally, all relevant potential strong confounding factors should be identified. The actual operational definition of these requirements will need to be on a case-by-case basis depending on the specific exposure–response relation studied.

### Criteria related to the quality of assessment and assignment of exposure of HOS

#### Is exposure expressed on a ratio scale and specific for the agent of interest?

If exposure is expressed on a ratio scale, the units of the scale represent the same magnitude of exposure across the whole range of the scale, and a rational zero is included (Stevens 1946). Quantitative exposure measurements, therefore, should be at the basis of exposure assessment. HOS that present quantitative exposure estimates based solely on expert judgment should not be used in QRA because of difficulties with regard to calibration of these estimates. For QRA, the exposure measures reported in HOS need to be specific for the agent of interest. Only a highly specific measure of exposure can be used to demonstrate a potential causal relation between exposure and health effect.

#### Quality of the exposure measurement methods

Quantitative measurements used in the exposure assessment in HOS can potentially differ with regard to the quality of the measurement methods and the analytical methods used. A guideline to evaluate HOS based on the quality of exposure measurements is to compare the method(s) used in the study to the method(s) that are currently considered as best practice. Some studies provide information on side-by-side comparisons of the exposure measurement method used with the best practice at the time of the study. Additional information from studies that focus solely on side-by-side comparisons of exposure measurement methods can be used as well (de Vocht et al. 2006; Stephenson et al. 2004).

#### Insight in the variability of exposure

For the evaluation of HOS, it is important to realize that exposure measurements used in exposure assessment can be highly variable in level. This variability can be attributed to a combination of variation in exposure levels over time and space. Advanced methodologies to acquire insight in the level of measurement variability on HOS outcomes have been proposed (Heederik and Miller 1988; Kromhout et al. 1999; Loomis et al. 1998; Xue et al. 2006). Before the evaluation, risk assessors must define a minimum acceptable level of information required to assess whether enough insight in variability of exposure measurements is provided in HOS. Tielemans et al. (2002) have developed guidelines to evaluate exposure data from HOS performed in the occupational exposure context. Similar approaches should be applied to exposure data from other exposure contexts (e.g., dietary exposure, consumer exposure). Differences between HOS in the ability to assess the relative contribution of the different sources of variability in exposure measurements can be used to rank the HOS.

#### Application of exposure measurements in exposure assessment

In most HOS, researchers are confronted with a scarcity of exposure measurements. As a result, exposure measurements might not be available for each assignment unit (i.e., a single individual or a group of individuals with assumed similar exposure patterns) for the complete time period of interest. In this situation, exposure measurements performed for assignment unit–time period combinations and information regarding the circumstances of these measurements (e.g., year of measurement, type of weather during measurement, or the task the measured individual performed during the measurement) are used to estimate exposure levels for assignment unit–time period combinations for which exposure measurements are not available. The strategy used to extrapolate measurements over assignment unit–time period combinations determines the validity of the exposure estimates and therefore has a large impact on the overall quality of the quantification of exposure. In most HOS, exposure measurements are extrapolated following a set of decision rules based on expert judgment and/or via a modeling framework. A complete and detailed insight in the applied decision rules in these approaches is essential for evaluation of HOS.

#### Type of exposure metric

In an ideal situation, an exposure metric captures three aspects that determine exposure: intensity, duration, and timing (Vacek 1997). The quality of an exposure metric is based on biologic considerations such as the time window of exposure that is relevant to the health effect of interest (Loomis et al. 1998; Seixas et al. 1993; Vacek 1997). A guideline to evaluate HOS based on the exposure metric used is to compare the metric used with the current state of knowledge on the nature of the relation between the exposure and health outcome of interest.

#### Specificity of the exposure indicator

In situations where it is difficult to assess the actual exposure that is assumed to be causally related to the health effect of interest, a causal indicator of exposure, researchers might assess a proxy for the causal exposure. However, it is crucial that the proxy exposure is highly correlated to the exposure of interest. Once absorbed in the human body, distribution, metabolism, and excretion have a large impact on the dose of a specific agent (or metabolite) at the site of action. Application of exposure indicators capable of incorporating these biologic influences in exposure estimates will result in increased correlation between the exposure indicator and the dose at the site of action. The application of biomarkers of exposure in HOS potentially provides the possibility to obtain exposure indicators with higher specificity compared with indicators of external exposure. Similar, as with external exposure, insight in variability of bio-marker-based exposure measurements is of utmost importance for QRA.

#### Blinded exposure assessment

Exposure assessment should always be performed blinded for the health outcome of interest to avoid observer bias. If exposure assessment was performed on the individual level, omission of a statement regarding blinded exposure assessment is a reason to exclude HOS from QRA. If exposure assessment was performed to assess exposure for previously defined homogeneous exposure categories, there is no direct connection between the individuals in the study population and the exposure assessment, and therefore this criterion needs less stringent application.

#### Quality of the exposure assignment strategy

In the exposure assignment step, exposure levels assessed for specific assignment unit–time period combinations are translated into exposure estimates for each individual in the study population. Assignment is based on information related to the individuals in the study population and related to the assignment unit–time period combinations for which exposure levels have been assessed. Examples of this information are the jobs an individual performed during his or her working career, a description of daily diet, or information on other factors potentially affecting exposure levels. The exposure context in which HOS are performed determines which type of information is available for exposure assignment. A proper evaluation of the quality of exposure assignment requires insight in the proportion of the assignment unit–time period combinations used for assignment for which no or little exposure measurements were available and exposure levels had to be inferred. In addition, the overlap between the assignment unit–time period combinations for which exposure measurements were available and the exposure time periods that are assumed to be relevant to the assessed health risk needs to be evaluated.

## Application of the Guidelines on Benzene Case–Control and Cohort Studies

### Selection of studies eligible for evaluation

**Figure 2:**
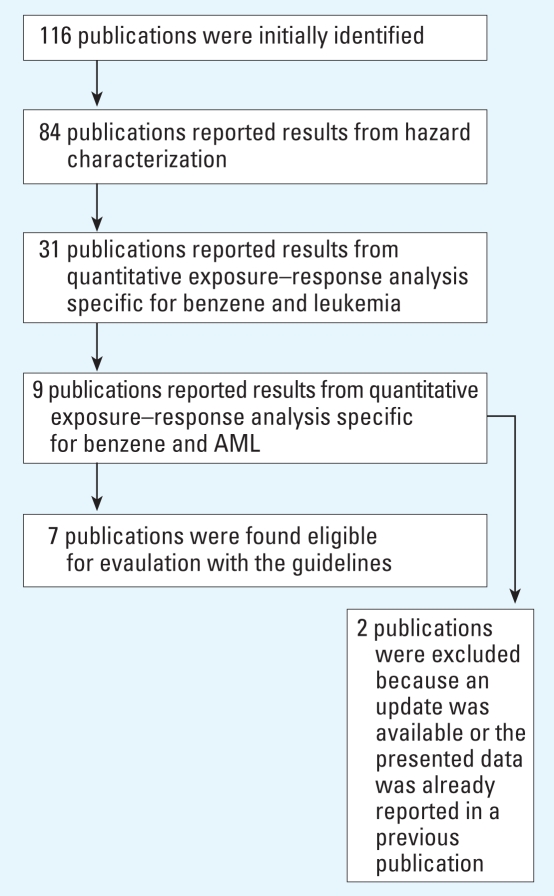
Overview of the strategy that was applied to select publications that report on the relation between exposure to benzene and AML and are eligible for evaluation with the guidelines.

To test the usefulness and practical implications of our guidelines, we applied the developed framework to all case–control and cohort studies that have reported on a dose–response relation between exposure to benzene and acute nonlymphocytic leukemia (ANLL) or AML. In this example we will ignore the small differences in disease classification between ANLL and AML and consider both as the same health outcome (referred to as AML). A detailed report of the selection of publications that were eligible for evaluation is presented in the Supplemental Material [see Supplemental Material II, Search Strategy (http://www.ehponline.org/members/2008/11530/suppl.pdf) for details]. All identified publications were reviewed for eligibility of application of the evaluation guidelines (Figure 2). Thirty-two publications were found not eligible because results from hazard characterization were not reported. From the 84 publications that did report results from hazard characterization, 53 publications were excluded because no quantitative exposure–response analysis specific for benzene and leukemia was reported. Finally, 22 publications did not report results from quantitative exposure–response analysis specific for benzene and AML. Therefore, the selection strategy resulted in only seven studies eligible for evaluation. Details of these studies are presented in Table 2.

### Evaluation

A detailed report of the evaluation is presented in the Supplemental Material [see Supplemental Material III, Outcome of the Evaluation (http://www.ehponline.org/members/2008/11530/suppl.pdf) for details]. Here we discuss the aspects that contributed to the ranking of the seven remaining HOS on benzene and AML that were evaluated with the use of our guidelines.

#### Definition of minimal requirements for QRA and identification of potential strong confounding factors

Before the evaluation, we defined minimal requirements for inclusion into QRA: response rate > 60%; loss to follow-up < 10%; and follow-up time > 10 years. We considered exposure to ionizing radiation as the only factor for which there is evidence of potential confounding on the relation between exposure to benzene and AML (Pagano et al. 2006).

#### Initial evaluation

Two studies, by Guénel (Guénel et al. 2002) and Monsanto (Collins et al. 2003; Ireland et al. 1997), did not pass the initial evaluation. The Guénel study was excluded because exposure was not presented on a ratio scale, but in unit-years (criterion 1.2). This limitation prohibits the use of this study in QRA, and therefore further evaluation was not done. The Monsanto study was excluded because of the very limited information that was provided on the statistical analysis performed (criterion 1.3). All other studies passed initial evaluation. It was assumed that exposure to ionizing radiation was not above background level in all the populations studied. Therefore, no potential strong confounding factors needed to be considered in the evaluation (criterion 1.6)

#### Categorization

From the studies that passed initial evaluation, two were case–control studies: AHW (Australian Health Watch) (Glass et al. 2000, 2003, 2005) and U.K. Petrol (Lewis et al. 1997; Rushton and Romaniuk 1997), and three were cohort studies: CAPM-NCI (Chinese Academy of Medicine–National Cancer Institute) (Dosemeci et al. 1994; Hayes et al. 1997; Travis et al. 1994; Yin et al. 1994), Dow (Bloemen et al. 2004; Ott et al. 1978), and Pliofilm (Paxton et al. 1994a, 1994b; Rinsky 1989; Rinsky et al. 1987; Wong 1995). The case–control studies were all nested in large occupational cohorts.

#### Design-specific evaluation

Design-specific criteria that contributed to the ranking based on quality were related to exposure assessment, exposure assignment, and insight in systematic error in exposure assessment/assignment. All studies (*n* = 5) reported the use of exposure measurements in the exposure assessment. However, there was a wide range in the amount of information that was provided regarding the quality of the measurements, insight in the variability of the measurements, and the use of measurements in exposure assessment. The AHW study and the U.K. Petrol study provided the most detailed information and apparently applied the most stringent quality criteria for inclusion of measurements in exposure assessment. The CAPM-NCI study reported the use of short-term area measurements but provided very little information regarding the quality and variability of these measurements. The Dow study reported that an industrial hygienist categorized all job titles into exposure categories that were defined in an earlier study on the same cohort with the use of industrial hygiene measurements. However, the actual relation between exposure measurements and exposure assessment is unclear. The Pliofilm study provided limited information on the measurements used in exposure assessment. However, it was reported that the measurements used for the Pliofilm cohort reflected benzene concentrations in workplace area and no personal sampling was performed. Exposure assignment strategy was most detailed in the U.K. Petrol, AHW, and CAPM-NCI studies. These studies reported the use of job- or task-specific and time-specific information for assignment. The Pliofilm study applied a less detailed assignment strategy based on a job title–exposure class matrix and provided limited insight in the exposure assignment strategy. Dow reported very limited information regarding assignment of exposure, which made proper evaluation impossible. Only one study performed a sensitivity analysis to acquire insight in the potential of systematic error due to potential biases such as misclassification of exposure and quality of work histories used in assignment (U.K. Petrol).

#### Ranking of the evaluated studies

Based on our evaluation, the two case–control studies, the U.K. Petrol and AHW studies, have received the highest relative ranking for QRA (Table 2). Although the study designs of the U.K. Petrol and AHW studies were comparable, the U.K. Petrol study was ranked higher because this study reported results from a sensitivity analysis used to evaluate the impact of several crucial decisions made in the assessment of exposure. The rationale to assign a lower ranking to the CAPM-NCI and the Pliofilm studies is that in both studies considerable uncertainty existed regarding the quality of the exposure measurements used and the methods used to incorporate exposure measurements in the assessment and assignment of exposures. The CAPM-NCI study provided more detailed information on the methods used for exposure assessment and was therefore ranked higher than the Pliofilm study. Although the Dow study was considered suitable for QRA, large uncertainty remained regarding the potential contribution of this study to QRA. This uncertainty was largely determined by the lack of information on the actual use of exposure measurements in assessment and assignment of exposure. Therefore the Dow study received the lowest ranking.

### Discussion of the application of the guidelines in the benzene–AML example

In our example, differentiation of the five studies suitable for QRA was based largely on the quality of assessment and assignment of exposure. In general, evaluation was difficult because of the limited information provided in the evaluated publications. Therefore, it is possible that the evaluation outcome of this example is based partly on the absence of information. Recently, the STROBE (Strengthening the Reporting of Observational Studies in Epidemiology) initiative provided general requirements for reporting of HOS (Vandenbroucke et al. 2007). Application of such requirements in the publication of studies will facilitate the evaluation of HOS. Unfortunately, STROBE proposes only limited guidelines for the reporting of exposure assessment in HOS and is therefore of limited use for the evaluation of HOS for QRA. In our example we evaluated only publications published in the peer-reviewed scientific literature. An alternative approach is to contact the researchers responsible for the studies selected for evaluation to acquire as much detailed information as possible. In our evaluation, each study included had specific limitations with regard to the quality of the estimation of quantitative exposure levels. As a result of this situation, several studies have been the subject of discussion regarding the quality and validity of exposure estimates (Paustenbach et al. 1992; Wong 1999). We think that a thorough sensitivity analysis that provides insight in the level of uncertainty of the estimated exposure levels and a detailed description of the approach used for assessment and assignment of exposure could have left less room for discussion and thereby would have increased the quality of all evaluated HOS for QRA. For the design of future quantitative HOS in this field, researchers should be aware of the specific requirements of QRA to HOS with regard to study design and reporting of results.

### Impact for human regulatory risk assessment of benzene

We compared the outcome of our evaluation with the selection of studies used in the regulatory QRA performed by the U.S. Environmental Protection Agency (EPA) in 1985 and updated in 1998 (U.S. EPA 1998). The U.S. EPA QRA is based on the study by Rinksy et al. (1987) (Pliofilm), Wong (1987), and Ott et al. (Bond et al. 1986; Ott et al. 1978) (Dow). A difference between the U.S. EPA QRA and our evaluation is the health end point that was considered. Whereas we evaluated only studies that reported specific risk estimates for AML, the U.S. EPA QRA focused on all leukemias together as a single health outcome. Therefore, the study by Wong (1987) was not considered in our evaluation because this study did not report specific risk estimates for AML. Based on our evaluation, three additional studies should be considered for a regulatory QRA of benzene: U.K. Petrol, AHW, and CAPM-NCI. Interestingly, these three studies were all regarded as providing higher quality evidence than the Pliofilm and the Dow study using our proposed framework. To assess the contribution of evidence from a single HOS to regulatory QRA, the assessment of the quality of the evidence needs to be combined with an assessment of the relevance of the evidence for QRA. The combination of quality and relevance of evidence is defined as the weight of evidence for QRA (Weed 2005). Aspects that contribute to the relevance of evidence for QRA are the exposure context in which the study was performed (e.g., occupational exposure vs. dietary exposure), the range of exposure levels included in the study, and the potential impact of random error on the study findings, usually quantified with confidence intervals (CIs). In Table 3 an overview of these aspects that contribute to the relevance of a study to QRA are presented for the five studies that we evaluated. In our example all included studies were performed in the occupational exposure context. However, the U.K. Petrol, AHW, CAPM-NCI, and Dow studies included ranges of benzene exposure levels that are thought to be more relevant for the current work population and the general population than the range of exposures that was included in the Pliofilm study (Table 3). Therefore, these studies require less extrapolation to calculate relevant risk estimates. To assess the potential impact of random error on the study findings, the fold range of the 95% CIs surrounding the relevant risk estimates is reported for each relevant risk estimate that was reported in the evaluated studies (Table 3). Relatively large differences in fold ranges were observed. We expect that a renewed QRA that included all quantitative epidemiologic evidence available at this time and incorporated a weight of evidence approach would significantly increase the confidence in unit risk estimates for exposure to benzene. Our approach contributes to a transparent qualitative insight in the differences in the weight of evidence of HOS for QRA. Quantification of the weight of evidence based on a review of the quality and the relevance of the available studies will be highly subjective and, if performed at all, should be as transparent as possible. Although existing approaches acknowledge the importance of exposure assessment in HOS for QRA (Goldbohm et al. 2006; Hertz-Picciotto 1995), we attempted to improve these methods by providing a detailed discussion of the aspects that collectively determine the quality of assessment and assignment of exposure in HOS. The outcome of the benzene–AML example indicated that, in this case, there were large differences between HOS with regard to the quality of the exposure assessment that would not have been detected with the application of the existing evaluation approaches.

## Figures and Tables

**Table 1:** Overview of the criteria used in the three-tiered evaluation of HOS for QRA.^a^

Tier	Evaluation criteria	Outcome	Impact on evaluation	CC^b^	COH^c^	CR^d^
I^e^	1.1 Is the study design case–control, cohort, or cross-sectional?	Yes/no	Selection for QRA^f^	X	X	X
I^e^	1.2 Is exposure expressed on a ratio scale and specific for the agent of interest?	Yes/no	Selection for QRA^f^	X	X	X
I^e^	1.3 Is a detailed description of the statistical analysis provided?	Yes/no	Selection for QRA^f^	X	X	X
I^e^	1.4 Are criteria for inclusion of subjects into the study described with sufficient detail?	Yes/no	Selection for QRA^f^	X	X	X
I^e^	1.5 Is the assessment of the health effect performed according to recognized norms?	Yes/no	Selection for QRA^f^	X	X	X
I^e^	1.6 Are all relevant potential strong confounding factors considered in the study design?	Yes/no	Selection for QRA^f^	X	X	X
II^g^	2.1 Type of study design	Case–control/cohort/cross-sectional	Selection for QRA^f^/study quality ranking^h^	X	X	X
III^i^	3.1 Response rate	Numerical	Selection for QRA^f^/study quality ranking^h^	X	X	X
III^i^	3.2 Loss to follow-up	Numerical	Selection for QRA^f^/study quality ranking^h^		X	
III^i^	3.3 Minimum follow-up time	Description	Selection for QRA^f^		X	
III^i^	3.4 Quality of the exposure measurement methods	Description	Selection for QRA^f^/study quality ranking^h^	X	X	X
III^i^	3.5 Insight in the variability of exposure	Description	Study quality ranking^h^	X	X	X
III^i^	3.6 Application of exposure measurements in exposure assessment	Description	Selection for QRA^f^/study quality ranking^h^	X	X	X
III^i^	3.7 Type of exposure metric	Description	Study quality ranking h	X	X	X
III^i^	3.8 Specificity of the exposure indicator	Category^j^	Study quality ranking h	X	X	X
III^i^	3.9 Blinded exposure assessment	Description	Selection for QRA^f^	X	X	X
III^i^	3.10 Quality of the exposure assignment strategy	Description	Study quality ranking h	X	X	
III^i^	3.11 Potential for information bias	Description	Study quality ranking h	X	X	X
III^i^	3.12 Blinded health outcome assessment?	Description	Selection for QRA^f^		X	X
III^i^	3.13 Insight in the potential for systematic error in study results	Description	Study quality ranking h	X	X	X

a Evaluation criteria are discussed in detail in Supplemental Material I.

b Criteria relevant for case–control (CC) study design.

c Criteria relevant for cohort study (COH) design.

d Criteria relevant for cross-sectional study (CR) design.

e Tier I: initial evaluation.

f Criteria relevant for selection of HOS for QRA.

g Tier II: categorization of HOS into three types of study designs that can potentially be used in QRA.

h Criteria relevant for ranking of studies based on quality of design, conduct, and reporting.

i Tier III: specific evaluation of the quality of the design, conduct, and reporting of HOS.

j Categories are constructed based on a combination of proxy vs. causal exposure and external vs. internal exposure.

**Table 2:** Summary details of the quantitative benzene–AML case–control and cohort studies ranked based on the outcome of the evaluation.

Ranking based on evaluation of study quality	Name of the study	Type of study design	Publications used for evaluation	Date of publication of hazard characterization	Evaluation outcomes that contributed to the differentiation of the evaluated HOS
1	U.K. Petrol^a^	Nested case–control	Lewis et al. 1997; Rushton and Romaniuk 1997	1997	+ Detailed insight in methodology for assessment and assignment of exposures + Limitations of exposure measurements were assessed and discussed + Potential for systematic error was assessed
2	AHW^b^	Nested case–control	Glass et al. 2000, 2003, 2005	2003	+ Detailed insight in methodology for assessment and assignment of exposures + Limitations of exposure measurements were assessed and discussed − Potential for systematic error was not assessed
3	CAPM-NCI^c^	Cohort	Dosemeci et al. 1994; Hayes et al. 1997; Travis et al. 1994; Yin et al. 1994	1997	+ Insight in methodology for assessment and assignment of exposure − Limited insight in quality and use of exposure measurements
4	Pliofilm^d^	Cohort	Paxton et al. 1994a, 1994b; Rinsky 1989; Rinsky et al. 1987; Wong and Raabe 1995	1995	+ Insight in methodology for assessment and assignment of exposure − Limited insight in quality and use of exposure measurements
5	Dow^e^	Cohort	Bloemen et al. 2004; Ott et al. 1978	2004	− Limited insight in methodology for assessment and assignment of exposure − Actual use of exposure measurements in exposure assessment is unclear
—	Guénel^f^	Nested case–control	Guénel et al. 2002	2002	Study not suitable for QRA
—	Monsanto^g^	Cohort	Collins et al. 2003; Ireland et al. 1997	2003	Study not suitable for QRA

+, positive study aspect; −, negative study aspect.

a Study performed on petroleum distribution workers in United Kingdom

b Australian Health Watch study.

c Study performed by Chinese Academy of Preventive Medicine (CAPM) and the U.S National Cancer Institute (NCI).

d Study performed on workers employed at two Ohio factories producing hydrochloride.

e Study performed on Dow Chemical Michigan Operations employees.

f Study performed by Guénel et al. on men employed at EDF-GDF.

g Study performed on Monsanto plant employees.

**Table 3:** Aspects that contribute to the relevance of HOS to regulatory QRA.

Name of study	Exposure context in which the study was performed	Size of the study population	Exposure categories included in study (ppm-years)^a^	Fold range of the 95% CIs reported for relevant risk estimates^b^
U.K. Petrol	Occupational exposure	31 cases/121 controls	0.26–0.59	14.6
			0.60–1.64	13.3
			1.65–4.78	13.2
			≥4.79	13.4
AHW	Occupational exposure	11 cases/44 controls	4–8	100.0
			> 8	31.8
CAPM-NCI	Occupational exposure	110,633 individuals (21 cases)	< 40	14.0
			40–99	14.5
			≥100	10.5
Pliofilm	Occupational exposure	1,868 individuals (6 cases)	< 40	221
			40–200	—c
			200–400	29.9
			> 400	14.2
Dow	Occupational exposure	2,266 individuals (4 cases)	< 28.3	28.5
			28.3–79.1	204.3
			> 79.1	223.8

a Exposure categories for which a risk estimate was reported for AML in the evaluated publications.

b Fold range was calculated as (upper bound of the 95% CI) / (lower bound of the 95% CI) for each exposure group for which a risk estimate was reported for AML in the evaluated publications.

c No cases were observed in this study for this exposure category; therefore, the lower bound of the 95% CI was 0 and a fold range could not be calculated.
